# Ketogenic Diet in Super-Refractory Status Epilepticus: A Retrospective Cohort Study with Severity-Matched Controls in Critically Ill Adults

**DOI:** 10.1007/s12028-025-02431-w

**Published:** 2026-01-08

**Authors:** Katharina Feil, Sophia Kindzierski, Constanze Single, Lena Geiger-Primo, Daniela Schweikert, Michael Adolph, Josua Kegele, Holger Lerche, Ulf Ziemann, Leona Möller, Annerose Mengel, IGNITE Study Group

**Affiliations:** 1 Department of Neurology and Stroke, University of Tübingen, Tübingen, Germany; 2 Hertie Institute for Clinical Brain Research, University of Tübingen, Tübingen, Germany; 3 Nutrition Support Team, University Medicine Tübingen, Tübingen, Germany; 4 Department of Anesthesiology and Intensive Care Medicine, University Hospital Tübingen, Tübingen, Germany; 5 Department of Neurology and Epileptology, University of Tübingen, Tübingen, Germany; 6 Department of Neurology, Epilepsy Center Hessen, Philipps University Marburg, Marburg, Germany; 7 Neurology, University of Ulm, Ulm, Germany

**Keywords:** Super-refractory status epilepticus, Ketogenic diet, Neurointensive care, Seizure control, Nonpharmacologic therapy

## Abstract

**Background:**

Super-refractory status epilepticus (SRSE) is a life-threatening neurological emergency with limited treatment options. A ketogenic diet (KD) is increasingly considered as a rescue therapy, but controlled data in critically ill adults remain scarce. This study aimed to evaluate the feasibility, safety, and clinical effects of KD in adult SRSE using a severity-matched control group.

**Methods:**

A retrospective, severity-matched cohort study compared adult patients with SRSE treated with KD to matched controls. The primary outcome was SRSE resolution. Secondary outcomes included the modified Rankin Scale (mRS) and mortality at 3 and 6 months. Time-dependent and multivariate Cox regression models adjusted for illness severity (including age and Status Epilepticus Severity Score [STESS]) and delayed KD initiation. Despite pragmatic matching, baseline differences in age, STESS, and seizure type were addressed through multivariate adjustment.

**Results:**

A total of 34 adult patients with SRSE were analyzed (18 KD, 16 control). KD was initiated after a mean of 16.6 ± 9.4 days and maintained for 12.9 ± 7.7 days. Ketosis was achieved in 33%, with mild, manageable complications in 28%. SRSE resolution occurred in 61.1% of KD patients vs. 87.5% of controls (*p* = 0.125), although KD patients had significantly longer status epilepticus duration and higher medication burden. Time-dependent Cox regression showed an association with faster SRSE resolution after KD initiation (hazard ratio [HR] 0.26, 95% confidence interval [CI] 0.07–0.97; *p* = 0.045). In the multivariate Cox model, KD remained independently associated with SRSE resolution (HR 0.368, 95% CI 0.176–0.756; *p* = 0.006). Earlier KD initiation was independently associated with improved seizure control (*p* = 0.015). At 3 and 6 months, KD patients showed significantly better functional outcomes (*p* = 0.023 and *p* = 0.021, respectively). Ketosis or ketone levels were not associated with outcome, suggesting that therapeutic effects may be independent of measurable ketosis.

**Conclusions:**

KD is feasible and safe in adult patients with SRSE. Time-dependent models showed a significant therapeutic association, particularly with earlier initiation. These findings support prospective evaluation of KD as a nonpharmacologic therapy in neurocritical care.

**Supplementary Information:**

The online version contains supplementary material available at 10.1007/s12028-025-02431-w.

## Introduction

Super-refractory status epilepticus (SRSE) is defined as status epilepticus (SE) persisting or recurring ≥ 24 h after initiation of anesthetic therapy. It is associated with high morbidity and mortality and represents one of the most challenging neurological emergencies in adult neurological intensive care units (ICUs) [1]. Although SE and refractory SE (RSE) often respond to escalating pharmacologic treatments, SRSE represents a distinct clinical entity requiring prolonged anesthetic treatment, intensive care management, and nonstandard therapeutic approaches [2]. Despite established protocols for early-stage treatment of SE, therapeutic strategies in SRSE often rely on low-level evidence such as case series or small retrospective cohorts [2–6]. If immunologic mechanisms are suspected, immunotherapies such as corticosteroids, intravenous immunoglobulin, or plasma exchange are often employed. In addition, therapeutic rescue strategies frequently include adjustment or intensification of antiseizure medications (ASMs), followed by off-label use of third-line ASMs (e.g., perampanel, stiripentol), hypothermia, neurosurgical resection, neuromodulation (e.g., vagus nerve or deep brain stimulation, electroconvulsive therapy, transcranial magnetic stimulation), and metabolic interventions such as the ketogenic diet (KD) [5]. KD, a high-fat, low-carbohydrate metabolic therapy, originally established in pediatric epilepsy, has gained increasing attention as an adjunctive treatment for SRSE in both children and adults [7–11]. In pediatric populations, KD has shown high efficacy, particularly in new-onset refractory status epilepticus (NORSE) and febrile infection–related epilepsy syndrome, with reported seizure control rates ranging from 67 to 88% [12, 13]. In adult ICU patients, the evidence is more limited but growing: early reports include a case of intravenous KD initiation in a critically ill patient [10], followed by a 2014 multicenter retrospective study that reported resolution of SRSE within a median of 3 days after KD initiation in nine of ten adult patients [9]. A prospective multicenter phase I/II trial of enteral KD in 15 adults with SRSE demonstrated a 73% resolution rate, with ketosis achieved in a median of 2 days and a mortality rate of 33% [14]. A single-center feasibility study evaluated 11 adult ICU patients with RSE treated with KD. Ketosis was achieved in 91% of patients within one day, and seizure resolution occurred in 73%. Although 27% developed SRSE, the study demonstrated overall feasibility, safety, and therapeutic potential of early KD initiation in critically ill adults [11]. An exploratory retrospective cohort study of 140 adults with RSE, including 32 treated with KD, found that KD was associated with seizure cessation in 81% and moderated the discharge modified Rankin Scale (mRS) in patients with higher age and seizure severity. The findings suggest a neuroprotective effect of KD in patients with severe RSE [15]. A comprehensive review and pooled analysis compiled data from 75 studies, including 276 patients (208 pediatric and 68 adult) with SRSE treated with KD. The overall KD response rate was 71.4%, achieved after a median of 6.5 days. Among patients with NORSE (*n* = 182), response was slightly lower at 64.3%, with a longer latency to effect (median 8 days). Prior epilepsy, known etiology, and fewer prior therapies were associated with better outcomes. Adverse effects occurred in 44.9%, with the most frequent being metabolic disturbances [13]. A prospective single-center study on 12 patients reported a 75% resolution rate in patients with SRSE following KD initiation [7]. However, the cohort mainly consisted of adolescents and young adults with cryptogenic NORSE, lacking the demographic and clinical complexity typically encountered in adult ICU patients, such as older age, multimorbidity, prolonged mechanical ventilation, and systemic complications. Recent reviews and guidelines acknowledge the potential of KD in adult patients with SRSE but emphasize the limited number and quality of studies, the frequent lack of control groups, and the underrepresentation of typical ICU patients [5, 16].

To address these gaps, we conducted a retrospective, severity-matched single-center cohort study comparing KD-treated patients with SRSE with standard care controls. This study reports real-world data on the feasibility, metabolic effects, seizure control, and clinical outcomes of KD in adult patients with SRSE under routine neurocritical care conditions, using case-severity-matched controls used for descriptive comparison.

## Methods

### Study Design and Setting

We conducted a retrospective, single-center cohort study at the Department of Neurology, University Hospital Tübingen, Germany. The study was approved by the local ethics committee (No. 069/2024BO2). It included adult patients admitted to the neurological ICU between January 2019 and December 2023 who fulfilled criteria for SRSE.

### Definition of SRSE

SRSE was defined as SE persisting or recurring ≥ 24 h after the initiation of continuous intravenous anesthetic therapy or recurring after attempted withdrawal of anesthesia, in line with current international definitions [1].

### Patient Selection and Data Collection

Patients were identified through systematic screening of all SRSE cases treated in the neurological ICU between 2019 and 2023 using the electronic ICU database. Inclusion criteria were as follows: (1) age ≥ 18 years, (2) diagnosis of SRSE, and (3) availability of detailed clinical and outcome data. Exclusion criteria included incomplete documentation, predefined limitations of treatment at admission, and alternative diagnoses. Between 2019 and 2023, 417 ICU admissions were screened, corresponding to 389 unique patients with SE (*International Classification of Diseases, Tenth Revision* code G41.x). After exclusion of 30 patients with predefined treatment limitations, 359 remained eligible for analysis. Of these, 180 fulfilled criteria for RSE and 79 fulfilled criteria for SRSE. Eighteen patients with SRSE received KD and were included in the intervention group, and 55 served as potential non-KD controls. The control group was retrospectively assembled from the same ICU population using a pragmatic severity-matching approach based on key clinical parameters, including age, sex, SRSE etiology, premorbid mRS (pmRS), and SE duration. Due to the small and clinically heterogeneous SRSE population, exact matching across all variables was not feasible. In particular, SE duration was substantially longer in KD-treated patients, reflecting the delayed and selective use of KD in prolonged and highly refractory cases. Residual imbalances—particularly in age, Status Epilepticus Severity Score (STESS), and seizure type—were addressed through multivariate and time-dependent regression models. STESS represents clinical severity at SE onset and does not reflect subsequent treatment refractoriness or prolonged ICU course. Consequently, temporal and clinical differences between groups remained despite pragmatic matching, consistent with real-world therapeutic decision-making in SRSE. The final matched cohort comprised 34 adult patients with SRSE (18 KD, 16 controls). The complete patient screening, exclusion, and inclusion process is summarized in the patient flowchart (Supplementary Fig. 1).

### Clinical and Severity Scoring

SE severity was assessed using the STESS [17]. Global illness severity at ICU admission was quantified using the Simplified Acute Physiology Score II (SAPS II) [18]. Level of consciousness was evaluated using the Glasgow Coma Scale (GCS) at ICU admission and discharge as well as at the beginning and ending of KD. Functional outcome was measured using the mRS at ICU discharge and at 3 and 6 months’ follow-up based on structured documentation and follow-up visits [19]. Functional good outcome was defined as an mRS ≤ 2 (https://eso-stroke.org/outcome-measures-stroke-modified-rankin-scale-ordinal-logistic-regression). Treatment limitations were defined as restrictions of life-sustaining treatment based on poor neurological prognosis, patient will, or surrogate decision. These included withholding or withdrawal of critical care interventions such as escalation of anesthetic therapy, reintubation, or cardiopulmonary resuscitation, either predefined or decided during the clinical course.

### KD Protocol

KD was administered exclusively enterally according to a standardized institutional protocol introduced in 2019 using a 4:1 fat-to-nonfat ratio. Serum β-hydroxybutyrate levels were monitored regularly (intended at least daily) to document onset and maintenance of ketosis. Further protocol details are published elsewhere [20].

### Outcomes

The primary outcome was SRSE resolution, defined as sustained clinical and electrographic seizure cessation for at least 24 h after anesthetic withdrawal without recurrence. Secondary outcomes comprised time to SRSE resolution, time from SE onset to KD initiation, time to ketosis, and maximum serum β-hydroxybutyrate concentration. Further outcomes included ICU stay, ventilation and hospital stay duration, GCS dynamics, systemic complications, KD-related adverse effects, premature KD discontinuation, functional status (mRS at discharge, 3 months, and 6 months), and mortality.

### Statistical Analysis

Descriptive statistics were used to summarize baseline characteristics. Continuous variables were compared using the Mann–Whitney *U*-test, and categorical variables were compared using Fisher’s exact test or the χ^2^ test. Time-to-event data (i.e., time from status onset to SRSE resolution) were analyzed using Kaplan–Meier survival analysis with log-rank testing to compare KD and non-KD groups. Additionally, we performed Cox proportional hazards regression, including both standard and time-dependent models, to evaluate the association between KD initiation and probability of SRSE resolution. The time-dependent model accounted for the delayed initiation of KD relative to SE onset and included KD as a time-varying covariate. Covariates in adjusted models included age, sex, STESS, premorbid mRS, and the cumulative number of ASMs and anesthetics administered. In a subset of patients treated with KD, we also explored the association of KD timing, ketosis achievement, and maximum ketone levels with the likelihood of SRSE termination. These exploratory models included univariate and multivariate Cox regression. To assess model stability and address the limited sample size, bootstrap resampling (2,000 iterations) was performed for selected Cox models. Model performance was evaluated using concordance (Harrell’s C) and standard diagnostic tests (likelihood ratio, Wald, and score tests). Missing data were handled using listwise deletion for regression models and are reported accordingly. Multivariate binary logistic regression analyses were performed to explore potential predictors of SRSE resolution. Independent variables included treatment with KD (yes/no), duration of SE, and cumulative number of ASMs and anesthetics. Model performance was evaluated using standard diagnostics. Due to the small sample size, confidence intervals (CIs) and *p* values were additionally estimated using bootstrap resampling (2,000 samples, percentile method). The number of events per variable was monitored to avoid overfitting. Logistic regression models for good functional outcome were not feasible due to complete or quasi-complete separation (i.e., all favorable outcomes occurred in KD-treated patients). Analyses were conducted using SPSS (IBM Corp., version 29) and R (R Core Team, version 4.3.2), with a significance threshold set at *p* < 0.05.

## Results

### Cohort Characteristics

A total of 34 critically ill adult ICU patients with SRSE were included: 18 patients received KD, and 16 served as severity-matched controls. KD patients tended to be younger (57.7 ± 18.0 vs. 67.2 ± 8.6 years, *p* = 0.063) and had lower STESS (3.3 ± 1.8 vs. 4.5 ± 1.0, *p* = 0.025). Seizure type distribution differed significantly, with more nonconvulsive SE in controls (*p* = 0.005). Sex, premorbid status, SAPS II, and comorbidities were otherwise balanced (Table 1).

**Table 1:** Baseline characteristics in the KD and control groups

Variable	KD group (*n* = 18)	Control group (*n* = 16)	*p* value
Age (years), mean ± SD	57.7 ± 18.0	67.2 ± 8.6	0.063
Sex (female), *n* (%)	8 (44.4%)	9 (56.2%)	0.732
pmRS, median (IQR)	0 (0–3)	0 (0–0)	0.122
Etiology of SE, *n* (%)			0.098
Structural (AIS, ICH, TBI, brain tumor, post-anoxic)	5 (27.8%)	9 (56.3%)	
Nonstructural (autoimmune, infectious, genetic, NORSE, others)	13 (72.2%)	7 (43.8%)	
Type of seizures, *n* (%)			0.005*
focal	1 (5.6%)	0 (0%)	
focal nonaware	7 (38.9%)	1 (6.3%)	
generalized	4 (22.2%)	3 (18.8%)	
nonconvulsive	6 (33.3%)	12 (75.0%)	
STESS, mean ± SD	3.3 ± 1.8	4.5 ± 1.0	0.025*
GCS at admission, mean ± SD	8.3 ± 5.3	7.1 ± 4.9	0.591
Ischemic stroke, *n* (%)	3 (16.7%)	1 (6.3%)	0.600
ICH or SAH, *n* (%)	1 (5.6%)	3 (18.8%)	0.320
Prior malignancy, *n* (%)	3 (16.7%)	5 (31.3%)	0.407
Diabetes mellitus, *n* (%)	1 (5.6%)	5 (31.3%)	0.068
Myocardial infarction, *n* (%)	4 (22.2%)	2 (12.5%)	0.064
Preexisting mild cognitive impairment, *n* (%)	3 (16.7%)	6 (37.5%)	0.180
Preexisting epilepsy, *n* (%)	6 (33.3%)	1 (6.3%)	0.090
Pathological cerebral imaging, *n* (%)	13 (72.2%)	14 (87.5%)	0.390
Contrast enhancement in imaging, *n* (%)	4 (22.2%)	2 (12.5%)	0.653
Cerebral atrophy, *n* (%)	4 (22.2%)	2 (12.5%)	0.653
Lumbar puncture performed, *n* (%)	11 (61.1%)	12 (75.0%)	0.476
Lumbar puncture, pathologic, *n* (%)	5 (27.8%)	5 (31.3%)	0.100
Cell count, lumbar puncture (cells/µL), mean ± SD	13.2 ± 16.5	10.6 ± 19.9	0.688
Lactate (mmol/L), lumbar puncture, mean ± SD	59.3 ± 44.2	56.0 ± 47.4	0.858
Protein, lumbar puncture (mg/dL), mean ± SD	2.0 ± 2.0	1.7 ± 1.3	0.642
Any limitations of treatment (DNR/DNI), *n* (%)	6 (33.3%)	3 (18.8%)	0.351

Abbreviations: AIS, acute ischemic stroke, DNI, do-not-intubate, DNR, do-not-resuscitate, GCS, Glasgow Coma Scale, ICH, intracerebral hemorrhage, IQR, interquartile range, KD, ketogenic diet, NORSE, new-onset refractory status epilepticus, pmRS, premorbid modified Rankin Scale, SAH, subarachnoid hemorrhage, SE, status epilepticus, STESS, Status Epilepticus Severity Score, TBI, traumatic brain injury

^*^Statistically significant (*p* < 0.05)

### Primary Outcome: SRSE Resolution and Disease Course

**Fig. 1:**
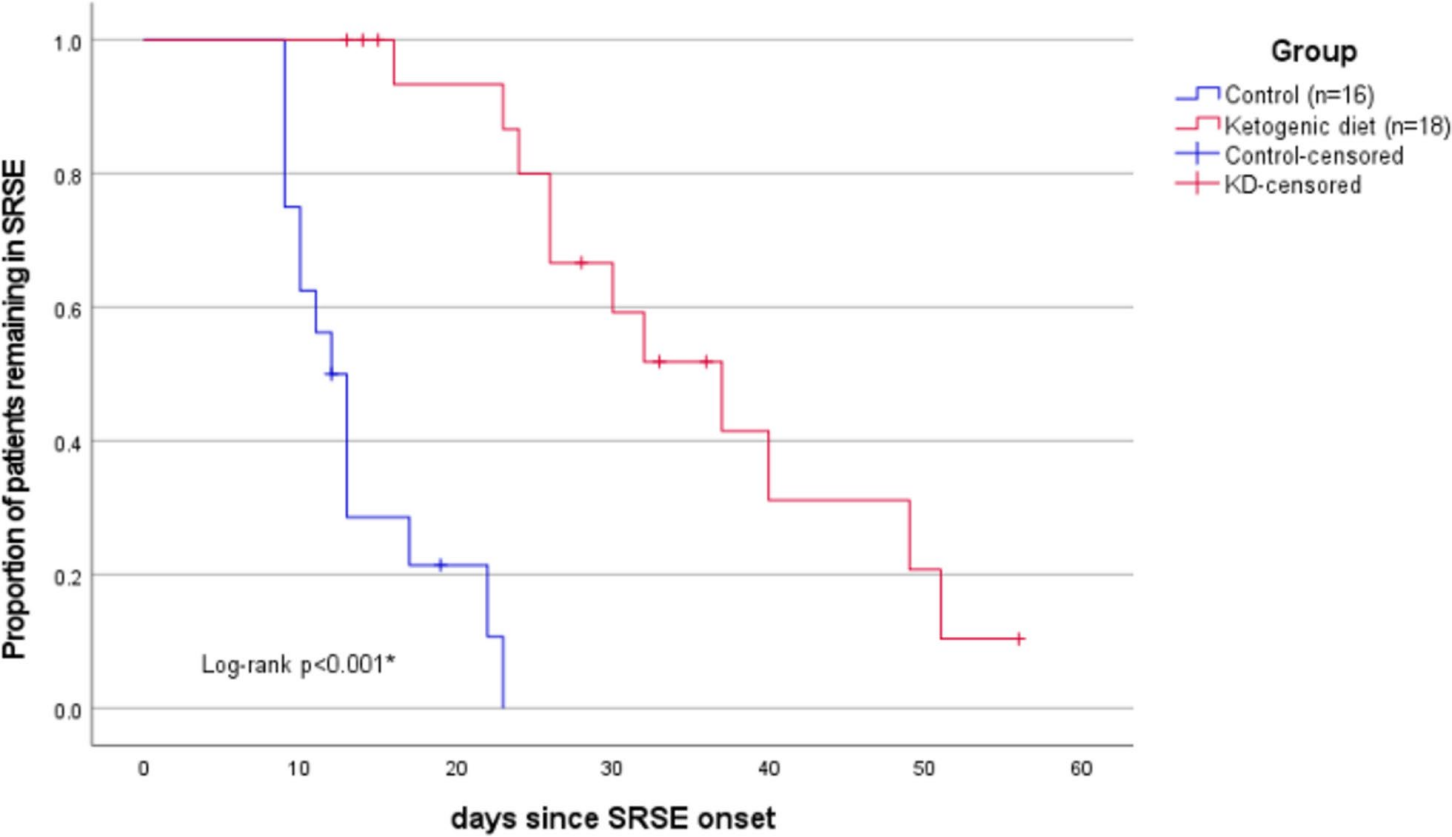
Kaplan–Meier analysis of time to SRSE resolution in patients with and without KD. Kaplan–Meier curve showing the proportion of patients remaining in SRSE over time. Patients treated with KD (red) had significantly longer SRSE durations compared to matched controls (blue), reflecting delayed treatment initiation and treatment selection bias. Censored cases (patients with no observed SRSE resolution due to therapy limitation, early death, or observation end) are indicated by tick marks. Log-rank *p* < 0.001. KD ketogenic diet, SRSE super-refractory status epilepticus

**Fig. 2:**
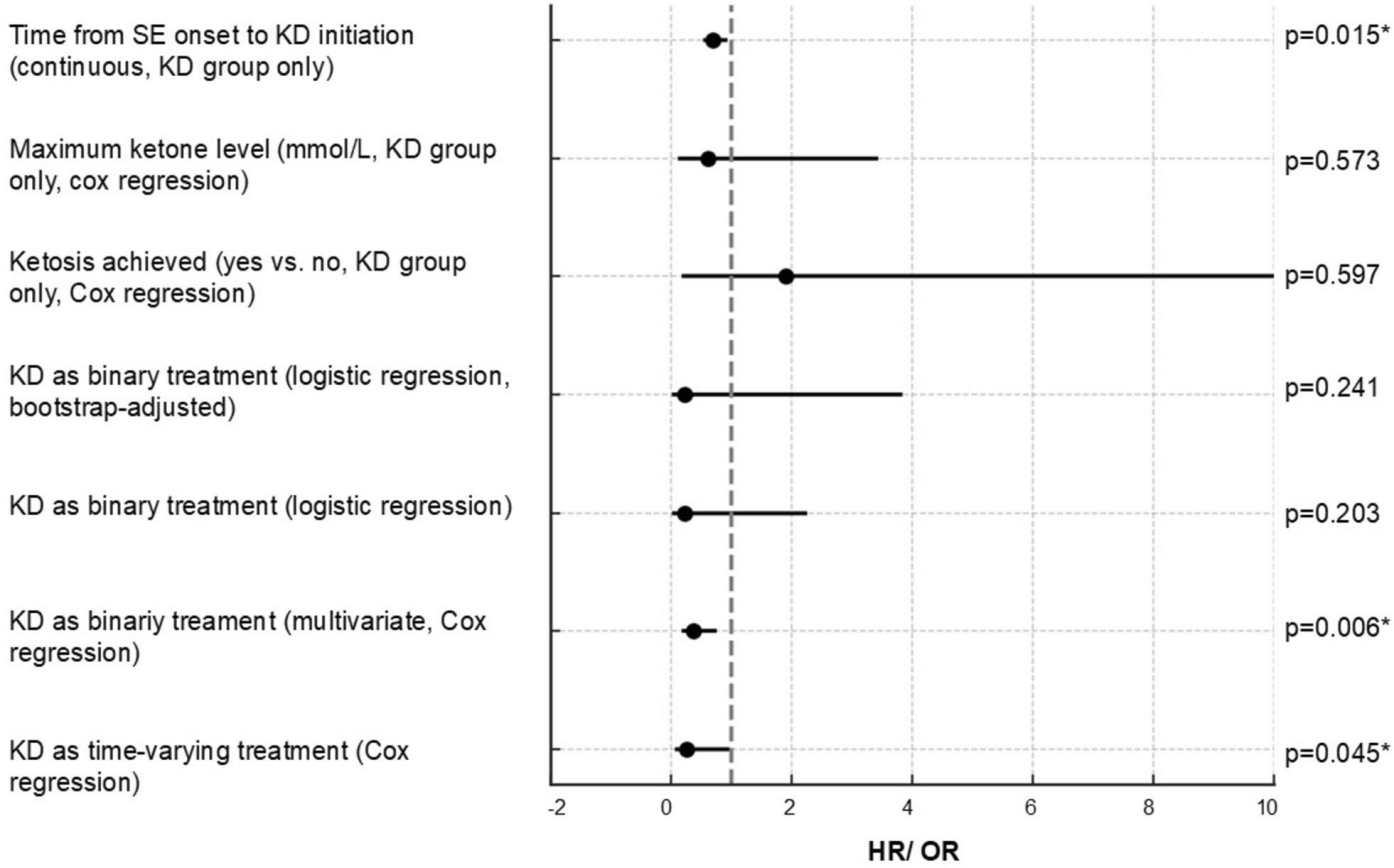
Association of KD and related predictors with SRSE resolution (event defined as persistence of SRSE). HRs and ORs are displayed from time-dependent and multivariate Cox regressions, as well as logistic regression models, including analyses restricted to KD-treated patients. Predictors included KD treatment (binary and time-dependent), timing of KD initiation, and markers of metabolic response (ketosis, ketone levels). Significant results (*p* < 0.05) are indicated by an asterisk. HR hazard ratio, KD ketogenic diet, OR odds ratio, SRSE super-refractory status epilepticus

SRSE resolution was achieved in 61.1% of KD-treated patients and 87.5% of controls (*p* = 0.125). However, SE duration was significantly longer in the KD group (30.5 ± 12.7 vs. 13.2 ± 4.6 days, *p* < 0.001), reflecting the delayed initiation of KD (mean 16.6 ± 9.4 days after SE onset). Kaplan–Meier analysis showed a significantly prolonged SRSE duration in KD-treated patients (log-rank *p* < 0.001) (Fig. 1). In contrast, time-dependent Cox regression showed KD was associated with significantly reduced hazard of ongoing SRSE (hazard ratio [HR] 0.26, 95% CI 0.07–0.97, *p* = 0.045), indicating faster resolution following KD initiation (Fig. 2). More cumulative ASMs/anesthetics were also independently linked to delayed resolution (HR 0.43, 95% CI 0.28–0.65, *p* < 0.001), likely reflecting disease severity. Age showed a trend-level effect (HR 0.96, 95% CI 0.92–1.00, *p* = 0.051) (Table 2).

**Table 2:** Results of time-dependent Cox regression model for super-refractory status epilepticus resolution

Variable	HR	95% CI	*p* value
KD (time-varying)	0.26	0.07–0.97	0.045*
Age (years)	0.96	0.92–1.00	0.051
Sex (female)	0.55	0.22–1.42	0.220
STESS	1.30	0.92–1.85	0.138
pmRS	0.81	0.48–1.39	0.450
Number of ASMs/anesthetics	0.43	0.28–0.65	< 0.001*

Abbreviations: ASM, antiseizure medication, CI, confidence interval, HR, hazard ratio, KD, ketogenic diet, pmRS, premorbid modified Rankin Scale, STESS, Status Epilepticus Severity Score

^*^Statistically significant (*p* < 0.05)

This analysis was complemented by a multivariate Cox model adjusting for baseline prognostic factors and treatment intensity. KD remained independently associated with SRSE resolution (HR 0.368, 95% CI 0.176–0.756, *p* = 0.006), confirming potential therapeutic benefit. Cumulative number of ASMs/anesthetics again predicted delayed resolution (HR 0.846, 95% CI 0.719–0.983, *p* = 0.029), whereas age, STESS, and premorbid mRS were not significant predictors (Table 3).

**Table 3:** Multivariate Cox regression on time to super-refractory status epilepticus resolution (2,000 bootstrap samples)

Variable	Hazard ratio	95% confidence interval	Bootstrap *p* value
KD	0.368	0.176–0.756	0.006*
ASMs/anesthetics (total)	0.846	0.719–0.983	0.029*
STESS	0.942	0.746–1.173	0.600
Age (years)	1.008	0.972–1.047	0.610
Premorbid mRS	0.711	0.456–1.102	0.120

Abbreviations: ASM, antiseizure medication, KD, ketogenic diet, mRS, modified Rankin Scale, STESS, Status Epilepticus Severity Score

^*^Statistically significant (*p* < 0.05)

### Secondary Outcome: Functional Status and Mortality

ICU stay (48.6 ± 41.1 vs. 30.7 ± 12.4 days, *p* = 0.093), hospital stay (56.6 ± 47.7 vs. 40.0 ± 16.4 days, *p* = 0.134), and ventilation duration (39.6 ± 40.6 vs. 24.6 ± 8.8 days, *p* = 0.142) were longer in KD patients, but not statistically significant. Burst suppression was more frequent in KD patients (66.7% vs. 37.5%, *p* = 0.047).

GCS score at discharge was similar (10.5 ± 5.1 vs. 9.5 ± 4.6, *p* = 0.507). Functional outcome tended to be worse in controls (median mRS 5 [interquartile range (IQR) 5–6] vs. 6 [IQR 5–6], *p* = 0.334). Favorable outcome (mRS score ≤ 2) occurred in one control and none of the KD group (*p* = 0.290). Mortality at discharge was lower in the KD patients (33.3% vs. 50.0%, *p* = 0.713).

At 3 months, functional outcomes differed significantly (median mRS score 5 [IQR 4–6] vs. 6 [IQR 6–6], *p* = 0.023), with favorable outcome in 16.7% of KD patients vs. 0% in controls (*p* = 0.209; 7 lost to follow-up). Mortality was lower in the KD group (33.3% vs. 50.0%, *p* = 0.005). At 6 months, KD patients had better mRS scores (median 5 [IQR 2–6] vs. 6 [IQR 6–6], *p* = 0.021), favorable outcome (27.8% vs. 0%, *p* = 0.086), and lower mortality (38.9% vs. 50.0%, *p* = 0.012) (Table 4).

**Table 4:** Outcome and clinical course (KD vs. control)

Variable	KD group	Control group	*p* value
SRSE solved (overall), *n* (%)	11 (61.1%)	14 (87.5%)	0.125
SRSE solved during KD regimen, *n* (%)	6 (33.4%)	–	–
Duration of SE (days), mean ± SD	30.5 ± 12.7	13.2 ± 4.6	< 0.001*
Burst suppression, *n* (%)	12 (66.7%)	6 (37.5%)	0.047*
Duration of invasive ventilation (days), mean ± SD	39.6 ± 40.6	24.6 ± 8.8	0.142
Length of ICU stay (days), mean ± SD	48.6 ± 41.1	30.7 ± 12.5	0.093
Length of hospital stay (days), mean ± SD	56.6 ± 47.7	40.0 ± 16.4	0.134
GCS at discharge, mean ± SD	10.5 ± 5.1	9.5 ± 4.6	0.507
Mortality at discharge, *n* (%)	6 (33.3%)	8 (50.0%)	0.713
Discharge destination, *n* (%)			0.429
Rehabilitation	4 (66.7%)	7 (87.5%)	
Home		1 (12.5%)	
mRS at discharge, median (IQR)	5 (5–6)	6 (5–6)	0.334
Good outcome at discharge, *n* (%)	0 (0%)	1 (6%)	0.290
mRS at 3 months, median (IQR)	5 (4–6)	6 (6–6)	0.023*
Good outcome at 3 months, *n* (%)	3 (16.7%)	0 (0%) (*n* = 7 missing)	0.209
Mortality at 3 months, *n* (%)	6 (33.3%)	8 (50.0%) (*n* = 7 missing)	0.005*
mRS at 6 months, median (IQR)	5 (2–6)	6 (6–6)	0.021*
Good outcome at 6 months, *n* (%)	5 (27.8%)	0 (0%) (*n* = 7 missing)	0.086
Mortality at 6 months, *n* (%)	7 (38.9%)	8 (50.0%) (*n* = 7 missing)	0.012*

Abbreviations: GCS, Glasgow Coma Scale, ICU, intensive care unit, IQR, interquartile range, KD, ketogenic diet, mRS, modified Rankin Scale, SE, status epilepticus, SRSE, super-refractory status epilepticus

^*^Statistically significant (*p* < 0.05).

### Treatment Characteristics, Feasibility, and Safety of KD

KD was initiated after a mean delay of 16.6 ± 9.4 days from SE onset and was maintained for 12.9 ± 7.7 days. Ketosis was achieved in 6 of 18 patients (33.3%) after 6.2 ± 3.2 days, with a mean maximum β-hydroxybutyrate level of 1.2 ± 1.0 mmol/L. Among KD-treated patients, the type and number of ASMs and anesthetics did not differ between those who achieved ketosis and those who did not, indicating that ASM profiles were comparable across groups. SRSE resolved during KD in six patients (33.3%). KD was discontinued in 77.8% of cases due to transition to palliative care (33.3%), other clinical decisions (33.3%), failure to achieve ketosis (22.2%), or adverse effects (5.6%). Side effects occurred in two patients (11.1%), and no severe metabolic complications were observed.

Neurological status improved under KD, with GCS scores increasing from 3.0 ± 0.0 to 5.4 ± 4.5. Laboratory parameters remained acceptable (Table 5). KD patients received more ASMs/anesthetics than controls (9.8 ± 2.0 vs. 6.6 ± 1.7, *p* < 0.001), consistent with higher refractoriness. They were also more likely to receive continuous benzodiazepines (66.7% vs. 25.0%, *p* = 0.038) (Supplementary Table 1).

**Table 5:** Feasibility and safety of KD

Variable	Value
Ketosis achieved, *n* (%)	6 (33.3%)
Time to ketosis (days), mean ± SD	6.2 ± 3.2
Maximum ketone level (mmol/L), mean ± SD	1.2 ± 1.0
Duration of KD (days), mean ± SD	12.9 ± 7.5
Duration of SE until beginning of KD (days), mean ± SD	16.6 ± 9.4
Duration KD regimen (days), mean ± SD	12.9 ± 7.7
Side effects observed, *n* (%)	2 (11.1%)
Reasons for KD termination, *n* (%)	
Palliative care	6 (33.3%)
Others	6 (33.3%)
Ketosis not reached	4 (22.2%)
Side effects	1 (5.6%)
SE resolved during KD, *n* (%)	6 (33.3%)
GCS at start of KD, mean ± SD	3.0 ± 0.0
GCS at end of KD, mean ± SD	5.4 ± 4.5
Laboratory parameters during KD, median (IQR)	
Blood glucose min (mg/dL)	79.5 (68.0–88.3)
Blood glucose max (mg/dL)	222.5 (166.5–309.0)
Triglycerides min (mg/dL)	144.0 (100.5–215.0)
Triglycerides max (mg/dL)	331.5 (161.8–687.5)
Cholesterol min (mg/dL)	116.5 (89.0–139.0)
Cholesterol max (mg/dL)	131.5 (103.3–167.8)
Sodium min (mmol/L)	136.0 (134.0–137.3)
Sodium max (mmol/L)	146.0 (143.0–151.0)
CRP max (mg/dL)	21.0 (13.0–30.0)
CK max (mg/dL)	203.0 (35.0–319.0)
Creatinine max (mg/dL)	0.9 (0.58–1.6)
Albumin (g/dL)	2.1 (1.9–2.2)

Abbreviations: CK, creatine kinase, CRP, C-reactive protein, GCS, Glasgow Coma Scale, IQR, interquartile range, KD, ketogenic diet, max, maximum, min, minimum, SE, status epilepticus

### Subgroup Analysis: KD Responders vs. Nonresponders

Among KD-treated patients, six were classified as responders (SRSE resolution during KD). Responders showed significantly greater neurological improvement (GCS score 9.8 ± 4.9 vs. 3.2 ± 1.6, *p* = 0.001), received fewer medications (7.4 ± 0.5 vs. 9.0 ± 1.5, *p* = 0.035), and had numerically better 6-month outcomes (mRS score 3 [IQR 2.0–5] vs. 6 [IQR 5–6], *p* = 0.079) (Table 6).

**Table 6:** KD patients stratified by SRSE resolution

Variable	SRSE solved (*n* = 11)	SRSE not solved (*n* = 7)	*p* value
Age (years), mean ± SD	55.5 ± 16.3	61.1 ± 21.2	0.529
Female sex, *n* (%)	4 (36.4%)	4 (57.1%)	0.417
Premorbid mRS, median (IQR)	0 (0–0)	3 (0–4)	0.026*
STESS, mean ± SD	3.2 ± 1.8	3.6 ± 1.9	0.665
Classification of seizures			0.583
Focal	1 (9.1%)	0 (0%)	
Focal nonaware	4 (26.4%)	3 (42.9%)	
Generalized	3 (27.3%)	1 (14.3%)	
Nonconvulsive	3 (27.3%)	3 (42.9%)	
Etiology of SE			0.337
Nonstructural (autoimmune, infectious, genetic, NORSE, others)	7 (63.6%)	6 (85.7%)	
Structural (AIS, ICH, TBI, brain tumor, post-anoxic)	4 (36.4%)	1 (14.3%)	
Known epilepsy, *n* (%)	2 (18.2%)	4 (57.1%)	0.097
GCS at admission, mean ± SD	8.4 ± 5.5	8.1 ± 5.5	0.935
Any limitations of treatment (DNR/DNI), *n* (%)	1 (9.1%)	5 (71.4%)	0.011*
Duration of SE (days), mean ± SD	32.2 ± 11.0	27.9 ± 15.6	0.499
Duration of hospital stay (days), mean ± SD	70.6 ± 45.8	34.6 ± 22.6	0.073
Duration of ICU stay (days), mean ± SD	59.7 ± 47.8	31.1 ± 20.0	0.156
Duration of mechanical ventilation (days), mean ± SD	49.2 ± 50.2	24.6 ± 5.9	0.220
Burst suppression, *n* (%)	7 (63.6%)	5 (71.4%)	0.751
Ketosis achieved, *n* (%)	4 (36.4%)	2 (28.6%)	0.751
GCS at start of KD, mean ± SD	3.0 ± 0.0	3.0 ± 0.0	1.000
GCS at end of KD, mean ± SD	6.8 ± 5.2	3.3 ± 0.8	0.094
Days to ketosis, mean ± SD	6.5 ± 3.6	4.5 ± 2.1	0.426
Max ketone level (mmol/L), mean ± SD	0.9 ± 0.7	0.8 ± 0.8	0.859
Max ketone level (mmol/L), median (IQR)	0.7 (0.7–1.5)	0.6 (0.4–1.1)	0.859
Side effects under KD, *n* (%)	1 (9.1%)	1 (14.3%)	0.751
Duration of KD (days), mean ± SD	14.3 ± 8.7	10.9 ± 4.9	0.360
SRSE solved during KD regimen	6 (54.5%)	0 (0%)	0.015*
ASMs/anesthetics total, mean ± SD	9.6 ± 1.3	10.1 ± 2.9	0.617
Number of ASM and anesthetics before KD, mean ± SD	7.8 ± 1.8	8.4 ± 2.7	0.574
Number of ASMs and anesthetics during KD, new, mean ± SD	2.0 ± 1.6	1.7 ± 1.4	0.711
GCS at discharge, mean ± SD	11.8 ± 4.6	4.0 ± 1.4	0.043*
mRS at discharge, median (IQR)	5 (5–5)	6 (5–6)	0.006*
Good outcome at discharge, *n* (%)	0 (0%)	0 (0%)	0.981
Mortality at discharge, *n* (%)	1 (9.1%)	5 (71.4%)	0.004*
mRS at 3 months, median (IQR)	4 (2–5)	6 (5–6)	0.010*
Good outcome at 3 months, *n* (%)	3 (27.3%)	0 (0%)	0.146
Mortality at 3 months, *n* (%)	1 (9.1%)	5 (71.4%)	0.004*
mRS at 6 months, median (IQR)	4 (2–5)	6 (5–6)	0.077
Good outcome at 6 months, *n* (%)	4 (36.4%)	1 (14.3%)	0.337
Mortality at 6 months, *n* (%)	2 (18.2%)	5 (71.4%)	0.023*

Abbreviations: ASM, antiseizure medication, DNI, do-not-intubate, DNR, do-not-resuscitate, GCS, Glasgow Coma Scale, ICU, intensive care unit, IQR, interquartile range, KD, ketogenic diet, max, maximum, mRS, modified Rankin Scale, SE, status epilepticus, SRSE, super-refractory status epilepticus, STESS, Status Epilepticus Severity Score

*Statistically significant (*p* < 0.05).

### Subgroup Analysis: Patients With vs. Without SRSE Resolution

In the total cohort, 25 patients achieved SRSE resolution, whereas 9 remained refractory. Responders had significantly lower premorbid disability (median pmRS score 0 [IQR 0–0] vs. 1 [IQR 0–4], *p* = 0.005) and fewer treatment limitations (16.0% vs. 55.6%, *p* = 0.034). Functional outcomes were better in responders at discharge (mRS score 5 [IQR 5–6] vs. 6 [IQR 5–6], *p* = 0.010), 3 months (mRS 5 [IQR 4–6] vs. 6 [IQR 6–6], *p* = 0.048), and 6 months (mRS score 5 [IQR 3–6] vs. 6 [IQR 6–6], *p* = 0.041). Mortality was lower at discharge (28.0% vs. 77.8%, *p* = 0.008) and 3 months (28.0% vs. 77.8%, *p* = 0.027), with a similar trend observed at 6 months (28.0% vs. 77.8%, *p* = 0.096). Other variables such as age, sex, structural vs. nonstructural etiology, or preexisting epilepsy did not differ. There were no significant differences in SE duration (21.4 ± 12.6 vs. 25.1 ± 14.7 days, *p* = 0.328), ICU stay, or imaging findings (Supplementary Table 2).

### Exploratory Analysis Within the KD Group

Within the KD group, 11 patients achieved SRSE resolution, whereas 7 remained refractory. Responders had lower premorbid disability (pmRS score 0 vs. 3, *p* = 0.026) and fewer treatment limitations (9.1% vs. 71.4%, *p* = 0.011). Functional and neurological outcomes were more favorable. No differences were observed in ketosis achievement, KD duration, or maximum ketone levels between those achieving resolution and those remaining refractory (Table 6).

In a separate analysis of patients with SRSE resolution during KD (*n* = 6), a lower cumulative medication burden (*p* = 0.035) and higher GCS score improvement (*p* = 0.001) were observed, whereas, again, all KD-associated factors did not differ (Supplementary Table 3).

In KD-treated patients, earlier KD initiation was significantly associated with SRSE resolution. In univariate Cox regression, each day of delay reduced the hazard of resolution by 12% (HR 0.881, 95% CI 0.800–0.970, *p* = 0.010). This was confirmed in a multivariate model adjusted for age, STESS, premorbid status, sex, and medication burden (HR 0.701, 95% CI 0.526–0.933, *p* = 0.015), suggesting a potential time-sensitive therapeutic window (Supplementary Table 4). Other variables (age, STESS, pmRS score, number of medications) were not significantly associated with outcome. To explore the role of ketosis, a Cox model using 36 KD treatment intervals found no significant effect of ketosis (HR 1.90, *p* = 0.597) or maximum ketone levels (HR 0.61, *p* = 0.573) on SRSE resolution (Supplementary Table 5).

In univariate logistic regressions, baseline variables (age, sex, body weight, SE etiology, time to KD) were not predictive of ketosis (all *p* > 0.2). These results suggest that KD timing may be more relevant than ketosis level. In a Cox regression comparing early vs. late KD (cutoff day 10), early KD showed a 3.6-fold higher hazard of resolution (HR 3.61, 95% CI 0.84–15.59, *p* = 0.085), supporting a time-sensitive effect despite limited power.

### Multivariate Analyses

In multivariate logistic regression including KD treatment, cumulative medication burden (ASMs and anesthetics), and SE duration, no variable reached significance. KD was not significantly associated with SRSE resolution (odds ratio [OR] 0.22, 95% CI 0.02–2.26, *p* = 0.203), although the effect direction suggested a possible protective trend. The wide CI indicates model instability and possible confounding due to delayed initiation (Supplement Table 6). An exploratory interaction term (KD × ASM burden) showed a nonsignificant trend (*p* = 0.128), possibly indicating enhanced KD efficacy in more refractory cases. Bootstrap-based CIs confirmed result instability, particularly for the KD effect, highlighting limitations of model robustness in this small cohort (Supplement Table 7).

## Discussion

Our retrospective severity-matched cohort study presents a comparative analysis between adult patients with SRSE treated with KD and a non-KD control group under routine neurocritical care conditions. Although not a randomized trial, our study constitutes the first comparative evaluation of KD use in adult patients with SRSE within a real-world ICU setting, applying severity-matched controls and time-dependent modeling to reduce bias. Our main findings are as follows: Firstly, our study confirms that KD is feasible and safe even in critically ill, treatment-refractory patients. It was initiated enterally after a median delay of > 2 weeks and maintained for ~ 13 days. Despite severe illness and concurrent intravenous therapies, ketosis was achieved in 33% of patients, with only mild side effects. The rate of ketosis is markedly lower than previously reported: Francis et al. reported 91% in a single-center RSE cohort [11], Cervenka et al. reported 73% in a prospective multicenter phase II trial of enteral KD in patients with SRSE [14], and Ren et al. reported 75% in patients with NORSE within a median of 3 days [7]. The lower rate may reflect delayed initiation, underlying metabolic instability, reduced enteral tolerance, and systemic complications. The use of a standardized 4:1 fat-to-nonfat protocol may have further contributed to delayed ketone accumulation compared to more flexible or individualized regimens such as the modified Atkins diet, an MCT-based diet, or parenteral formulations [9, 10]. However, no serious adverse events occurred, supporting KD as a viable adjunct even in complex ICU patients.

Secondly, SRSE resolved in 61.1% of KD-treated patients compared to 87.5% in the control group (*p* = 0.125). Although this unadjusted difference did not reach statistical significance and numerically favored controls, it likely reflects treatment selection and immortal time bias. KD was predominantly initiated in patients with longer and more refractory SE courses, as evidenced by significantly prolonged SE duration (30.5 ± 12.7 vs. 13.2 ± 4.6 days, *p* < 0.001), higher medication burden, and more frequent use of continuous anesthetics. Although the STESS was lower in the KD group, this score is known to have limited sensitivity in ICU cases and may not fully capture treatment refractoriness. This is consistent with prior findings showing that STESS has limited predictive value in ICU populations. Systemic illness and ICU-specific factors significantly impact outcomes, suggesting that SE scores require adaptation to better reflect the prognosis of critically ill patients [21]. In our cohort, surrogate markers such as prolonged SE duration, higher anesthetic load, and increased burst suppression more reliably reflected higher disease severity in the KD group compared to non-KD patients.

The resolution rate in our KD group was lower than in previous studies reporting 73–90% [7, 9, 14, 15], likely due to delayed initiation and greater refractoriness. On average, KD was started 16.6 days after SE onset, substantially later than in most prior studies (typically 1–3 days). KD patients received a mean of 9.8 ASM and anesthetic agents, consistent with advanced therapeutic exhaustion. Additional indicators of severity, such as higher frequency of burst suppression and longer ICU stay, further support this interpretation. An alternative hypothesis that KD may have delayed recovery or caused metabolic disturbances appears unlikely, given the time-dependent association with SRSE resolution and the low rate of mild, manageable complications.

Importantly, the time-dependent Cox regression model, which adjusts for immortal time bias, demonstrated a significantly reduced hazard of ongoing SRSE following KD initiation (HR 0.26, 95% CI 0.07–0.97, *p* = 0.045). This supports a potential therapeutic effect, even when used late. Furthermore, earlier KD initiation within the KD subgroup was independently associated with SRSE resolution (HR 0.70, 95% CI 0.53–0.93, *p* = 0.015), underscoring the importance of timing. Pediatric studies have reported similar time-dependent effects, linking early KD to faster seizure control and improved outcomes [22]. These findings support the hypothesis that KD may exert a delayed and cumulative therapeutic effect in SRSE rather than immediate seizure suppression. This aligns with metabolic remodeling and neuroprotective mechanisms hypothesized for KD, which may require several days to manifest clinically [16, 23, 24]. A review highlighted that although KD may reduce anesthetic requirements and facilitate weaning, full seizure cessation is rarely immediate and likely reflects delayed neuroprotective or antiinflammatory effects [16]. One pediatric study observed seizure control occurring 2 to 19 days after KD initiation, consistent with a gradual metabolic shift [24]. Other evidence suggests seizure control often follows prolonged SE and occurs several days after ketosis is established [23].

Thirdly, KD patients showed numerically better long-term outcomes and lower mortality, although not all comparisons reached significance. At 3 and 6 months, median mRS scores were significantly better in the KD group, with favorable outcomes (mRS score ≤ 2) in 16.7% and 27.8%, respectively, compared to 0% in controls. Six-month mortality was also lower (38.9% vs. 50%). Although follow-up losses and nonsignificant *p* values limit interpretation, these trends suggest KD may improve both seizure control and functional recovery. This association may reflect neuroprotective effects of ketosis or reduced sedative burden after SRSE resolution [5, 9]. Comparable results were reported by Koh et al., who found improved mRS scores in KD-treated patients with severe SE under continuous anesthesia [15]. Notably, treatment limitations (do-not-resuscitate (DNR)/do-not-intubate (DNI)) were more frequent in the KD group (33.3% vs. 18.8%, *p* = 0.351), suggesting that better outcomes were not due to less aggressive care.

Fourthly, in contrast to the association between KD initiation and SRSE resolution, a separate Cox model of 36 KD treatment intervals showed no significant association for ketosis (HR 1.90, *p* = 0.597) or peak ketone levels (HR 0.61, *p* = 0.573). In our cohort, ketosis did not reliably predict clinical response, suggesting it may reflect adherence rather than therapeutic efficacy. Multiple studies support the idea that KD response involves broader metabolic, inflammatory, and neuromodulatory effects. In Angelman syndrome, clinical improvement preceded measurable ketosis, indicating early-acting pathways [25]. In SRSE, higher baseline ketones predicted outcome, but treatment-phase levels did not correlate with seizure control [26]. Reviews emphasize KD’s multifactorial actions: antiinflammatory effects, mitochondrial optimization, neurotransmission modulation, and reduced oxidative stress [27]. These likely require sustained adaptation, not a specific ketone threshold. ICU-specific factors—fluid shifts, insulin, renal clearance, enteral absorption—further limit ketone interpretability. Thus, although ketosis confirms adherence, it may not linearly reflect efficacy. Our subgroup analysis further supports this, as ketosis achievement and KD duration did not differ between responders and nonresponders.

Fifthly, multivariate logistic regression failed to identify significant predictors of SRSE resolution. KD showed a nonsignificant association with reduced odds of resolution (OR 0.22), likely reflecting model instability and time-related confounding rather than a true detrimental effect. Bootstrap analyses confirmed result instability, particularly for the KD variable. In contrast, both time-dependent and multivariate Cox models consistently demonstrated a significant therapeutic association between KD and SRSE resolution, even after adjustment for illness severity and treatment intensity. The discrepancy highlights the limitations of logistic regression in small, nonrandomized cohorts with time-varying exposures. The absence of significant predictors likely reflects small sample size, few events, and clinical heterogeneity. Moreover, KD initiation was nonrandomized and influenced by prognosis and clinical judgment. Complete or quasi-complete separation (e.g., favorable outcomes only in KD patients) further hampered valid modeling. Such limitations are common in SRSE studies, where ethical and logistical barriers preclude randomized trials.

Taken together, our exploratory regression analyses suggest a potential therapeutic signal of KD in SRSE, yet results must be interpreted with caution. The observed trends—particularly the significant time-dependent association between KD and seizure resolution—underscore the need for prospective evaluation in larger, stratified, and ideally prospectively enrolled controlled studies.

This study has several limitations. First, it was a retrospective, single-center study with a small sample, limiting generalizability and statistical power. Despite severity-matching and multivariate adjustment, residual confounding cannot be excluded. Differences in age, STESS, and SE duration remained despite pragmatic matching. These reflect clinical reality rather than methodological bias, as KD was typically initiated in patients with prolonged and highly refractory SE who had already failed multiple treatments. STESS captures the initial severity at SE onset but not subsequent treatment refractoriness, which explains why KD patients appeared less severe by this score while representing the most refractory clinical subgroup. These imbalances were addressed through multivariate and time-dependent Cox regression models. Second, immortal time bias is inherent to observational studies with delayed interventions like KD; although addressed using time-dependent Cox models, selection effects may persist. Third, ketosis was not achieved in all patients, and variability in metabolic response may have influenced outcomes independently of KD efficacy. Fourth, long-term outcome data were incomplete in some patients due to loss to follow-up, particularly in seven control patients. Despite attempts to obtain information through follow-up letters and medical record queries, no data could be retrieved. These patients were often transferred to external facilities, and undocumented death cannot be excluded. Fifth, the study was not powered to detect rare complications or formally assess the impact of KD on recovery or mortality. Lastly, although SRSE resolution was defined using established criteria, interpretation of electroencephaloygraphy (EEG) or clinical improvement under sedation may have introduced classification bias.

This retrospective cohort study adds novel real-world data on KD use in adult patients with SRSE under routine neurocritical care conditions. Using a severity-matched control group and time-dependent Cox regression, we were able to explore potential therapeutic associations while accounting for delayed treatment initiation and illness severity. KD proved feasible and safe, even in severely ill, treatment-refractory patients. Although unadjusted analyses were affected by delayed KD initiation, time-dependent models demonstrated a significantly increased likelihood of SRSE resolution after KD. Importantly, earlier initiation was independently associated with improved seizure control. These findings suggest that KD should not be reserved as a last-resort therapy but rather considered earlier in the treatment course. Building on these results, a prospective multicenter study is currently being planned to implement the standardized KD protocol across neuro-ICUs in a pragmatic, stepped-wedge design, enabling structured evaluation of safety, feasibility, and clinical outcomes in adult patients with SRSE. This prospective design will help to determine optimal timing, efficacy, and effect on long-term outcomes and to define the role of KD in neurointensive care.

## Supplementary Information

Below is the link to the electronic supplementary material.

Supplementary file1 (DOCX 56 KB)

## Data Availability

The datasets generated and/or analyzed during the current study are available from the corresponding author on reasonable request.
