# Supplementary material for: Ketogenic Diet in Super-Refractory Status Epilepticus: A Retrospective Cohort Study with Severity-Matched Controls in Critically Ill Adults
Source: Neurocrit Care. 2026 Jan 8;44(3):875–87. doi: 10.1007/s12028-025-02431-w (PMC13249628; doi:10.1007/s12028-025-02431-w)
Supplement: Supplementary file 1 — Supplementary file1 (DOCX 56 KB) [file 12028_2025_2431_MOESM1_ESM.docx]

### **Supplementary:**

### Supplementary Figure 1: Flow chart of patient inclusion and cohort formation.


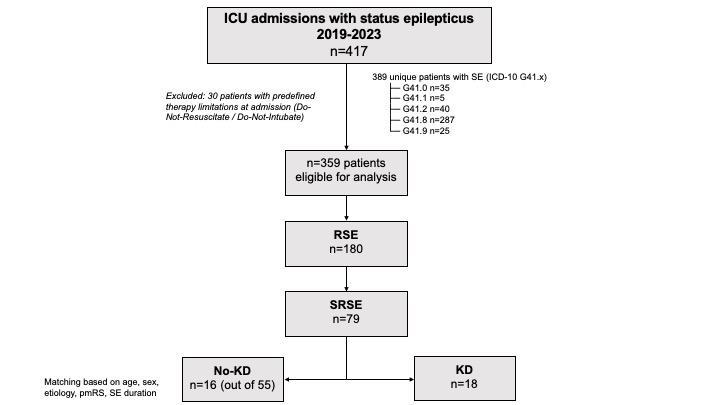


ICU admissions with status epilepticus (2019–2023, n = 417) were screened. After exclusion of patients with predefined therapy limitations (DNR/DNI), 359 were eligible for analysis. Of these, 79 fulfilled criteria for SRSE. Eighteen patients received KD and 16 matched non-KD controls were included in the final cohort. Abbreviations: KD: ketogenic diet, RSE: refractory status epilepticus, SRSE: superrefractory status epilepticus, ICU: intensive care unit

### Supplement Table 1. Antiseizure and anesthetic medication during SE

| **Variable** | **KD Group** | **Control Group (non-KD)** | **p-value** |
| --- | --- | --- | --- |
| **Total number of ASMs, mean±SD** | 6.8±1.4 | 4.2±1.2 | <0.001* |
| **Total number of ASMs, median (IQR)** | 7 (6, 8) | 4 (4, 5) | <0.001* |
| 4 ASMs, n (%) | 1 (5.6%) | 6 (37.5%) |  |
| 5 ASMs, n (%) | 2 (11.1%) | 5 (31.2%) |  |
| 6 ASMs, n (%) | 5 (27.8%) | 2 (12.5%) |  |
| 7 ASMs, n (%) | 4 (22.2%) | 1 (6.2%) |  |
| 8 ASMs, n (%) | 5 (27.8%) | 2 (12.5%) |  |
| 10 ASMs, n (%) | 1 (5.6%) | 0 (0.0%) |  |
| **Total number of anesthetic agents, mean±SD** | 3.1±0.9 | 2.3±1.0 | 0.011* |
| **Total number of anesthetic agents, median (IQR)** | 3 (3, 4) | 2 (2, 3) | 0.011* |
| **Total number of ASMs + anesthesia agents, mean±SD** | 9.8±2.0 | 6.6±1.7 | <0.001* |
| **Total number of ASMs + anesthesia agents, median (IQR)** | 10 (9, 11) | 6 (5, 8) | <0.001* |
| **New ASM during KD, mean±SD** | 1.5 ± 1.4 | – | / |
| **New ASM during KD, median (IQR)** | 1 (0, 3) | – |  |
| **New anesthesia agents during KD, mean±SD** | 0.4 ± 0.6 | – | / |
| **New anesthesia agents during KD, median (IQR)** | 0 (0, 1) | – |  |
| **New ASM + anesthesia agents during KD, mean±SD** | 1.9 ± 1.5 | – | / |
| **New ASM + anesthesia agents during KD, median (IQR)** | 2 (1, 3) | – |  |
| **ASM days brutto, mean±SD** | 156.1±92.0 | 69.3±32.6 | 0.001* |
| **ASM days netto, mean±SD** | 34.6±21.8 | 25.8±12.2 | 0.150 |
| **Anesthesia days brutto, mean±SD** | 71.9±43.3 | 35.8±17.0 | 0.003 |
| **Anesthesia days netto, mean±SD** | 31.4±13.3 | 20.1±7.3 | 0.004 |
| **Medication usage during SE, n (%)** |  |  |  |
| Levetiracetam | 14 (77.8%) | 16 (100.0%) | 0.105 |
| Valproate | 14 (77.8%) | 12 (75.0%) | 1.000 |
| Phenytoin | 12 (66.7%) | 11 (68.8%) | 1.000 |
| Phenobarbital | 5 (27.8%) | 2 (12.5%) | 0.405 |
| Lacosamide | 13 (72.2%) | 10 (62.5%) | 0.717 |
| Topiramate | 6 (33.3%) | 2 (12.5%) | 0.232 |
| Perampanel | 3 (16.7%) | 1 (6.3%) | 0.601 |
| Brivaracetam | 3 (16.7%) | 0 (0.0%) | 0.231 |
| Benzodiazepines | 18 (100.0%) | 16 (100.0%) | 1.000 |
| Benzodiazepine Perfusor | 12 (66.7%) | 4 (25.0%) | **0.038*** |
| Propofol | 15 (83.3%) | 12 (75.0%) | 0.695 |
| Ketamine | 8 (44.4%) | 6 (37.5%) | 0.728 |

Abbreviations: SD: standard deviation, ASM: antiseizure medication, IQR: interquartile range, KD: ketogenic diet, SE: status epilepticus

### **Supplementary Table 2**: Comparison of Patients with and without SRSE resolution (entire cohort)

| **Variable** | **SRSE solved n=25** | **SRSE not solved n=9** | **p-value** |
| --- | --- | --- | --- |
| Age (years) | 58.3 ± 17.3 | 64.8 ± 12.5 | 0.966 |
| Sex (female), n (%) | 9 (45.0%) | 8 (50.0%) | 0.257 |
| pmRS, median (IQR) | 0 (0, 0) | 1 (0, 4) | 0.005* |
| Etiology of SE   non-structurel, n (%)  structural, n (%) | 14 (56.0%) 11 (44.0%) | 6 (66.7%) 3 (33.3%) | 0.593 |
| STESS, mean±SD | 4.0±1.6 | 3.7±1.7 | 0.638 |
| Preexisting epilepsy, n (%) | 6 (30.0%) | 1 (6.3%) | 0.090 |
| Any therapy limitation | 4 (16.0%) | 5 (55.6%) | 0.034* |
| Ischemic stroke, n (%) | 3 (15.0%) | 1 (6.3%) | 0.614 |
| ICH or SAH, n (%) | 1 (5.0%) | 3 (18.8%) | 0.312 |
| Prior malignancy, n (%) | 3 (15.0%) | 5 (31.3%) | 0.407 |
| Diabetes mellitus, n (%) | 1 (5.0%) | 5 (31.3%) | 0.068 |
| Myocardial infarction, n (%) | 4 (20.0%) | 2 (12.5%) | 0.640 |
| Preexisting dementia, n (%) | 3 (15.0%) | 3 (18.8%) | 1.000 |
| Pathological imaging, n (%) | 14 (70.0%) | 13 (81.3%) | 0.695 |
| Cerebral imaging, contrast enhancement, n (%) | 4 (20.0%) | 2 (12.5%) | 0.653 |
| Cerebral atrophy, n (%) | 4 (20.0%) | 2 (12.5%) | 0.653 |
| GCS at admission, mean±SD | 8.1±5.1 | 7.0±5.3 | 0.580 |
| GCS at discharge, mean±SD | 10.8±4.6 | 4.0±1.4 | 0.057 |
| Duration of SE (days), mean±SD | 21.4±12.6 | 25.1±14.7 | 0.328 |
| Duration of mechanical ventilation (days), mean±SD | 35.4±35.4 | 24.6±5.1 | 0.113 |
| Length of ICU stay (days), mean±SD | 43.8±35.4 | 30.1±17.5 | 0.061 |
| Length of hospital stay (days), mean±SD | 50.3±41.3 | 33.9±19.7 | 0.169 |
| Burst suppression observed | 12 (48.0%) | 6 (66.7%) | 0.448 |
| KD regime, n (%) | 11 (44.0%) | 7 (77.8%) | 0.086 |
| Ketosis achieved, n (%) | 4 (36.4%) | 2 (28.6%) | 1.000 |
| KD terminated, n (%) | 3 (27.3%) | 3 (42.9%) | 0.621 |
| Duration of KD (days) | 11.2±4.2 | 12.6±4.3 | 0.513 |
| mRS at discharge, median (IQR) | 5 (5, 6) | 6 (5, 6) | 0.010* |
| Mortality at discharge, n (%) | 7 (28.0%) | 7 (77.8%) | 0.008* |
| Good outcome at discharge, n (%) | 1 (4.0%) | 0 (0%) | 0.557 |
| mRS at 3 months, median (IQR)  (n=7 missing) | 5 (4, 6) | 6 (6, 6) | 0.048* |
| Mortality at 3 months, n (%) | 7 (28.0%) | 7 (77.8%) | 0.027* |
| Good outcome at 3 months, n (%) | 3 (12.0%) | 0 (0%) | 0.502 |
| mRS at 6 months, median (IQR)  (n=7 missing) | 5 (3, 6) | 6 (6, 6) | 0.041* |
| Good outcome at 6 months, n (%) | 4 (16.0%) | 1 (11.0%) | 0.502 |
| Mortality at 6 months, n (%) | 7 (28.0%) | 7 (77.8%) | 0.096 |

Abbreviations: SE: status epilepticus, SRSE: uper-refractory status epilepticus, pmRS: premorbid modified Rankin Scale, STESS: Status Epilepticus Severity Score, ICH: intracerebral hemorrhage, SAH: subarachnoid hemorrhage, GCS: Glasgow Coma Scale, KD: ketogenic diet, mRS: modified Rankin Scale, ICU: intensive care unit, ASM: antiseizure medication, n: number, IQR: interquartile range, SD: standard deviation

### **Supplementary Table** 3: Comparison of KD Responders (SRSE resolution during KD) vs. Non-Responders

| **Variable** | **KD Responder (n = 6)** | **KD Non-Responder (n = 12)** | **p-value** |
| --- | --- | --- | --- |
| GCS at start of KD, mean ± SD | 3.0 ± 0.0 | 3.0 ± 0.0 | 1.000 |
| GCS at end of KD, mean ± SD | 9.8 ± 4.9 | 3.2 ± 1.6 | **0.001*** |
| mRS at discharge, median (IQR) | 5.0 (5.0, 5.0) | 5.0 (5.0, 6.0) | 0.318 |
| mRS at 3 months, median (IQR) | 4.0 (2.5, 4.8) | 5.0 (4.8, 6.0) | 0.109 |
| mRS at 6 months, median (IQR) | 2.5 (2.0, 4.5) | 5.5 (4.8, 6.0) | 0.079 |
| Ketosis achieved, n (%) | 5 (83.3%) | 7 (58.3%) | 0.592 |
| Days to ketosis, median (IQR) | 2.0 (1.0, 3.0) | 3.0 (2.0, 4.0) | 0.234 |
| Duration of mechanical ventilation, mean ± SD | 34.6±13.8 | 43.2±49.2 | 0.491 |
| Max ketone level (mmol/L), median (IQR) | 3.2 (2.4, 4.5) | 2.7 (1.7, 3.0) | 0.234 |
| Side effects under KD, n (%) | 1 (16.7%) | 3 (25.0%) | 1.000 |
| KD terminated, n (%) | 3 (50.0%) | 9 (75.0%) | 0.592 |
| Duration of KD (days), mean ± SD | 11.8 ± 3.2 | 12.6 ± 4.6 | 0.816 |
| Total number of ASMs, mean ± SD | 5.0 ± 0.0 | 5.9 ± 0.9 | 0.206 |
| Total anesthesia, mean ± SD | 2.4 ± 0.5 | 3.1 ± 0.9 | 0.326 |
| ASM + anesthesia total, mean ± SD | 7.4 ± 0.5 | 9.0 ± 1.5 | **0.035*** |
| Burst suppression, n (%) | 5 (83.3%) | 7 (58.3%) | 0.592 |
| Blood glucose min (mg/dL), median (IQR) | 63.0 (55.0, 77.0) | 65.0 (56.0, 76.0) | 0.911 |
| Blood glucose max (mg/dL), median (IQR) | 155.0 (133.0, 171.0) | 176.0 (157.0, 210.0) | 0.123 |
| Sodium min (mmol/L), median (IQR) | 133.0 (130.0, 134.0) | 134.0 (130.0, 135.0) | 0.816 |
| Sodium max (mmol/L), median (IQR) | 144.0 (143.0, 145.0) | 143.0 (140.0, 145.0) | 0.489 |
| CRP max (mg/dL), median (IQR) | 6.4 (5.4, 8.6) | 12.7 (6.6, 21.3) | 0.234 |
| CK max (U/L), median (IQR) | 227.0 (160.0, 439.0) | 241.0 (94.0, 1336.0) | 0.816 |
| Creatinine max (mg/dL), median (IQR) | 0.9 (0.8, 1.0) | 1.0 (0.8, 1.1) | 0.816 |

Abbreviations: KD: ketogenic diet, SD: standard deviation, IQR: interquartile range, ASM: antiseizure mediation, min: minimum, max: maximum, CRP: C-reactive protein, CK: creatine kinase.

### Supplementary Table 4. Multivariate Cox Regression in the KD-Group (n = 18)

| **Variable** | **HR** | **95% CI** | **p-value** |
| --- | --- | --- | --- |
| Number of ASM + anesthesia | 0.366 | 0.111 – 1.206 | 0.098 |
| STESS | 0.814 | 0.407 – 1.627 | 0.560 |
| Age (years) | 0.981 | 0.916 – 1.050 | 0.573 |
| pmRS | 0.564 | 0.239 – 1.335 | 0.193 |
| Female sex | 0.239 | 0.021 – 2.746 | 0.250 |
| Duration SE until KD start (days) | 0.701 | 0.526 – 0.933 | 0.015* |

Outcome: Time to SRSE resolution Note: The analysis was restricted to patients treated with KD. KD timing was included as a continuous predictor; all other covariates were included as shown. A HR < 1 indicates an association with faster SRSE resolution.

Abbreviations: KD: ketogenic diet, HR: hazard ratio, CI: confidence interval, ASM: Antiseizure medication; STESS: Status Epilepticus Severity Score, pmRS: premorbid modified Rankin Scale, SE: Status epilepticus, SRSE: Super-refractory status epilepticus.

### Supplementary Table 5. Cox Regression Analysis of Ketosis on SRSE Termination

| **Variable** | **HR** | **95% CI** | **p-value** |
| --- | --- | --- | --- |
| Ketosis (Yes vs No) | 1.90 | 0.18 – 20.67 | 0.597 |
| Max Ketone Level | 0.61 | 0.11 – 3.43 | 0.573 |

Model statistics: Concordance = 0.613 (SE = 0.088), Likelihood ratio test p= 0.8, Wald test p= 0.8, Log-rank test p= 0.8.

Abbreviations: HR: hazard ratio, CI: confidence interval, SE: Status epilepticus.

### **Supplementary Table 6. Multivariate logistic regression analysis for SRSE resolution**

Dependent variable: SRSE resolution (yes = 1, no = 0)

| **Variable** | **B** | **SE** | **Wald χ²** | **p-value** | **OR (Exp(B))** | **95% CI for OR** |
| --- | --- | --- | --- | --- | --- | --- |
| KD (yes vs. no) | –1.508 | 1.185 | 1.619 | 0.203 | 0.221 | 0.022-2.259 |
| Number of ASMs + anesthetics (total) | –0.247 | 0.260 | 0.906 | 0.341 | 0.781 | 0.469-1.300 |
| Duration of SE (days) | +0.035 | 0.043 | 0.651 | 0.420 | 1.035 | 0.952-1.126 |
| Constant | +3.368 | 2.030 | 2.752 | 0.097 | 29.023 | — |

Abbreviations: SRSE: Super-refractory status epilepticus, B: Regression coefficient (log odds), SE: Standard error; Wald χ²: Wald chi-square test statistic, OR: Odds ratio, CI: Confidence interval, ASM: Antiseizure medication, KD: ketogenic diet, SE: Status epilepticus

### **Supplementary Table 7. Bootstrap estimates for logistic regression model (2000 samples, percentile CI)**

| **Variable** | **B** | **Bias** | **SE (boot)** | **p-value (2-sided)** | **95% Bootstrap CI (percentile)** |
| --- | --- | --- | --- | --- | --- |
| KD (yes vs. no) | –1.508 | –1.476 | 8.198 | 0.241 | –21.859 to +3.836 |
| Number of ASMs + anesthetics (total) | –0.247 | –0.279 | 2.463 | 0.415 | –1.852 to +0.404 |
| Duration of SE (days) | +0.035 | +0.014 | 0.451 | 0.497 | –0.095 to +0.265 |
| Constant | +3.368 | +4.486 | 25.096 | 0.110 | –0.758 to +26.173 |

Abbreviations: B: regression coefficient (log odds), Bias = bootstrap bias estimate, SE (boot): bootstrap standard error, CI: confidence interval, ASM: antiseizure medication, SE = status epilepticus, KD: ketogenic diet
